# How do chiropractors manage clinical risk? A questionnaire study

**DOI:** 10.1186/2045-709X-21-18

**Published:** 2013-06-08

**Authors:** Martin Wangler, Cynthia Peterson, Beatrice Zaugg, Haymo Thiel, Rob Finch

**Affiliations:** 1 European Academy of Chiropractic, Bahnhofstrasse 15, Burgdorf, CH-3400, Switzerland; 2 University of Zürich and Orthopaedic University Hospital Balgrist, Forchstrasse 340, Zürich, 8008, Switzerland; 3 Swiss Academy for Chiropractic, Sulgenauweg 38, Bern, 3007, Switzerland; 4 ChiroSuisse, Eidgenössische Prüfung Chiropraktik, Johann-Verresisus Strasse 18, Biel, CH-2502, Switzerland; 5 Anglo‒European College of Chiropractic, 13-15 Parkwood Road, Bournemouth, BH5 2DF, UK; 6 The Royal College of Chiropractors, Chiltern Chambers, St Peters Avenue, Reading, RG4 7DH, UK

**Keywords:** Chiropractic, Safety, Risk, Incident-reporting

## Abstract

**Background:**

The literature on chiropractic safety tends to focus on adverse events and little is known about how chiropractors ensure safety and manage risk in the course of their daily practice. The purpose of this study was to investigate how chiropractors manage potentially risky clinical scenarios. We also sought to establish how chiropractors perceive the safety climate in their workplace and thus whether there is an observable culture of safety within the profession.

**Methods:**

An online questionnaire was designed to determine which of nine management options would be chosen by the respondent in response to four defined clinical case scenarios. Safety climate within the respondent’s practice setting was measured by seeking the level of agreement with 23 statements relating to six different safety dimensions. 260 licensed chiropractors in Switzerland and 1258 UK members of The Royal College of Chiropractors were invited to complete the questionnaire. Questionnaire responses were analysed quantitatively in respect of the four clinical scenarios and the nine management options to determine the likelihood of each option being undertaken, with results recorded in terms of % likelihood. Gender differences in response to the management options for each scenario were evaluated using the Mann–Whitney U (MWU) test. Positive agreement with elements comprising each of the six safety dimensions contributed to a composite ‘% positive agreement’ score calculated for each dimension.

**Results:**

Questionnaire responses were received from 76% (200/260) of Swiss participants and 31% (393/1258) of UK members of The Royal College of Chiropractors. There was a general trend for Swiss and UK chiropractors to manage clinical scenarios where treatment appears not to be successful, not indicated, possibly harmful or where a patient is apparently getting worse, by re-evaluating their care. Stopping treatment and/or incident reporting to a safety incident reporting and learning system were generally found to be unlikely courses of action. Gender differences were observed with female chiropractors appearing to be more risk averse.

**Conclusions:**

Swiss and UK chiropractors tend to manage potentially risky clinical scenarios by re-evaluating the case. The unlikeliness of safety incident reporting is probably due to a range of recognised barriers, although Swiss and UK chiropractors are positive about local communication and openness which are important tenets for safety incident reporting. The observed positivity towards key aspects of clinic safety indicates a developing safety culture within the Swiss and UK chiropractic professions.

## Background

It stands to reason that chiropractors aim to ensure their patients experience a comfortable and hazard-free environment, and provide treatment modalities that are safe. But do chiropractors routinely implement safety measures when managing patients? Clinical situations where safety would be expected to be an issue include:

•Treating a patient when it is not indicated.

•Treating a patient when it is *contra*-indicated.

•Failing to refer to another healthcare provider when required.

•Undertaking inappropriate/failing to undertake appropriate diagnostic tests.

•Continuing treatment when the patient is not getting better.

Any of the above could lead to injury or an undesired reaction, so it is important to be mindful of the risk of these situations arising.

The literature on safety in relation to chiropractic tends to focus on adverse events [1], and while safety incident reporting programmes are now available to the chiropractic profession in some countries as an educational risk-management measure [2], little is known about how chiropractors routinely ensure safety and manage risk in the course of their daily practice.

Our aim in the study reported here was to investigate how chiropractors manage potentially risky clinical scenarios. We did this by determining how chiropractors in Switzerland and the UK, where we had ready access to a population of chiropractors of reasonable size, would handle a range of defined clinical scenarios. These scenarios were devised to prompt reflection on patient management in the context of the safety considerations listed above, including whether to stop treatment (for reasons of safety) and/or report to a safety incident reporting and learning system. We also sought to establish how chiropractors perceive the safety climate in their workplace.

## Methods

### Questionnaire design and data collection

An anonymous, two-part, online questionnaire was developed using SurveyMonkey®. The first part of the questionnaire was designed to determine how respondents would handle four defined clinical scenarios, designed by us to prompt reflection on typical safety considerations. The scenarios were designed to be plausible and realistic and we tested this by seeking the views of a group of 16 experienced chiropractors. After adjustment, the final questionnaire included the four scenarios with the respondent able to choose from nine management options, with multiple answers possible, and provision for adding free-text comments. The nine management options were themed around either continuing, pausing or stopping treatment. We were interested to determine what the respondents felt was appropriate, and to look for consensus, without making any judgements ourselves.

The second part of the questionnaire, which was based closely on the validated Medical Office Survey on Patient Safety [3], aimed to determine how positive respondents were towards six safety climate dimensions within their clinic.

To ensure compliance with the Helsinki Declaration, the questionnaire was submitted to the Cantonal Research Ethics Committee (KEK), Bern, where it was confirmed to be ethically unproblematic due to its anonymous nature. A permit was not required. Questionnaire responses were retained in a SurveyMonkey® account which was password-protected in order to ensure data security. Potential respondents were assured of the complete anonymity of their responses and the investigators’ intention to publish the findings of their study. Submission of the completed questionnaire implied consent from the respondent.

The final version of the questionnaire listed the following four clinical scenarios:

1. A patient with non-specific low-back pain has not improved at all after 4–6 treatments.

2. A patient, who has a simple neck problem with no previous long-term problems, has now improved at least 80% and stayed at this level for a couple of weeks.

3. A patient returns from the last treatment with a new distal pain (e.g. sciatica when treated only for localized LBP, or brachialgia when treated only for local neck pain).

4. An elderly woman complains about immediate chest pain on inspiration after manual treatment directed to her thoracic spine.

The nine management options provided were as follows:

•I would re-evaluate the patient with a view to establishing a better diagnosis.

•I would send the patient for diagnostic imaging.

•I would change my treatment approach and use another technique.

•I would send the patient for a second opinion to another healthcare professional but keep on monitoring their condition.

•I would try a few times more.

•I would encourage the patient to continue the treatment until their spine is subluxation-free.

•I would stop treatment and monitor the patient regularly.

•I would stop the treatment, apologise and report the event to the chiropractic reporting and learning system.

•I would stop the treatment, but tell the patient that s/he is welcome to return if they feel the need.

Each of these management options had four different possible responses: ‘never’, ‘unlikely’, ‘likely’ and ‘most likely’. Respondents were also invited to provide comments on their choice from which we were particularly interested to identify views and attitudes in relation to safety incident reporting.

The second part of the questionnaire measured the safety climate within the practice setting by seeking the level of agreement on a five-point scale, with the responses ‘strongly disagree’, ‘disagree’, ‘neither agree nor disagree’, ‘agree’ and ‘strongly agree’, with 23 statements relating to six different safety dimensions, as follows:

•Teamwork – helping out, relationships, respect, teamwork-emphasis.

•Work pressure – rushing, overwork, staff contingent, patient numbers.

•Staff training – in response to new processes, on-the-job, appropriateness of tasks.

•Process and standardisation – organisation, procedures, workflow, processes.

•Communication openness – ideas for improvement, alternative views, asking questions, voicing disagreement.

•Patient tracking/follow-up – reminders, documentation, reports, monitoring.

The questionnaire also included a section to enable collection of the following demographic data: gender, age, college of graduation, number of years in practice and practice setting (alone or with others) to provide an opportunity to explore differences among groups within the responding cohort.

The link to the finalised online questionnaire was emailed to all 260 licensed chiropractors in Switzerland and 1258 UK members of The Royal College of Chiropractors. These Swiss and UK groups are each known to have access to a national online safety incident reporting and learning system (‘CIRLS’ [4] and ‘CPiRLS’ [5] respectively).

### Data analysis

The questionnaire responses were analysed quantitatively in respect of the four clinical scenarios and the nine management options described above. Management options identified as either ‘most likely’ or ‘likely’ by 75% or more of the respondents were arbitrarily designated, overall, as *likely* to be undertaken, and those options identified as either ‘most likely’ or ‘likely’ by 25% or fewer respondents were arbitrarily designated, overall, as *unlikely* to be undertaken. Thus, results were recorded in terms of ‘% likelihood’.

Positive agreement (strongly agree/agree with a positive statement or strongly disagree/disagree with a negative statement) with elements comprising each of the six safety dimensions contributed to a composite ‘% positive agreement’ score calculated for each dimension. A score greater than 60%, but less than 75%, was arbitrarily considered to indicate that respondents were *moderately positive* about the given safety dimension. A score of 75% or more was arbitrarily considered to indicate that respondents were *highly positive* about the given safety dimension.

Demographic differences in the responses to the management options for each of the four clinical scenarios were explored using the Mann–Whitney U (MWU) test [6]; P < 0.05 was considered statistically significant. The mean scores and standard deviations (SD) were also calculated for each management decision in these groups. SPSS version 17.0 was used for this data analysis.

We made general observations in respect of apparent differences in the responses from Swiss and UK-based chiropractors but did not statistically analyse these differences. Demographic differences were explored using a combined dataset.

## Results

### Response rate and demographic characteristics of respondents

The questionnaire response rate was 76% (200/260) for Swiss participants and 31% (393/1258) for UK participants.

The demographic data collected is shown in Table 1. In summary, among the Swiss responders there were more male responders than females (70:30) whereas, among the UK responders, the cohort was almost equally divided by gender. The most common age group for responders from both countries was 41–50 years. More than 90% of UK chiropractors had graduated from a UK institution, while all but 0.5% of respondents practising in Switzerland had qualified in the USA or Canada. In comparison to their UK colleagues, a greater proportion of those practising in Switzerland work on their own.

**Table 1:** Demographic details of the Swiss and UK respondents

**Respondent characteristics**		**Swiss respondents (n = 200)**	**UK respondents (n = 393)**
Gender	Male	70.0%	47.6%
	Female	30.0%	52.4%
Age group	21-30 years	2.5%	20.9%
	31-40 years	30.5%	27.5%
	41-50 years	37.0%	31.0%
	51-60 years	22.0%	15.5%
	61 years or above	8.0%	5.1%
College of graduation	Anglo-European College of Chiropractic	0.5%	51.6%
	McTimoney College of Chiropractic	0.0%	18.8%
	Welsh Institute of Chiropractic	0.0%	21.1%
	Other (Europe)	0.0%	0.0%
	Other (USA/Canada)	99.5%	8.5%
Number of years in practice	1-2 years	2.0%	13.5%
	3-5 years	9.5%	15.9%
	5-10 years	22.5%	21.9%
	10+ years	66.0%	48.7%
Practice setting	On your own	48.5%	32.8%
	With other chiropractor(s)	49.5%	66.2%
	With other health care practitioners	15.0%	37.9%

### Case scenarios

**Figure 1:**
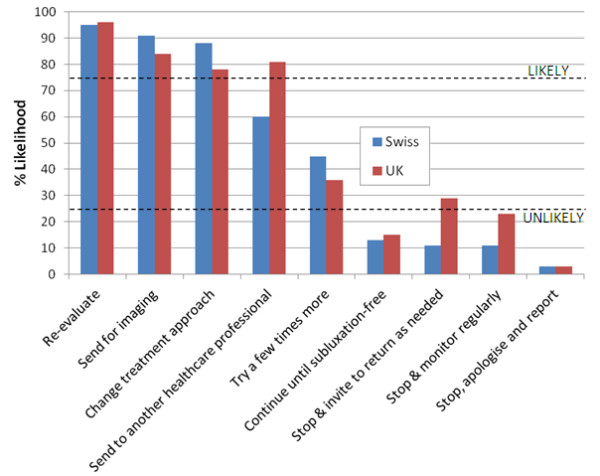
**Clinical management options in response to case scenario 1.** Likelihood of nine given clinical management options being followed in response to *Case Scenario 1*: *A patient with non*-*specific low*-*back pain has not improved at all after 4*–*6 treatments*. The dotted lines demarcate a region of the chart outside of which management options were arbitrarily designated as ‘likely’ (upper line) or ‘unlikely’ (lower line) to be undertaken. Thus, in this instance, Swiss and UK chiropractors are likely to re-evaluate, send for imaging, and change the treatment approach; UK chiropractors were likely to refer to another healthcare professional; both groups were unlikely to continue the treatment until the patient is subluxation-free, stop and then monitor regularly or stop to apologise and report to the incident reporting system; Swiss chiropractors were unlikely to stop and invite the patient to return as needed.

**Figure 2:**
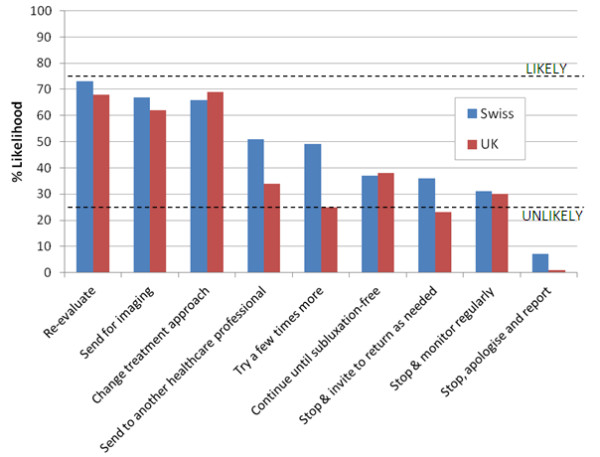
**Clinical management options in response to case scenario 2.** Likelihood of nine given clinical management options being followed in response to *Case Scenario 2*: *A patient*, *who has a simple neck problem with no previous long*-*term problems*, *has now improved at least 80*% *and stayed at this level for a couple of weeks*. The dotted lines demarcate a region of the chart outside of which management options were arbitrarily designated as ‘likely’ (upper line) or ‘unlikely’ (lower line) to be undertaken. In this instance, none of the management options were categorised by our criteria as *likely* by either Swiss or UK chiropractors; both groups were unlikely to stop to apologise and report to the incident reporting system; UK chiropractors were unlikely to try a few more times, or stop treatment and invite the patient to return as needed.

**Figure 3:**
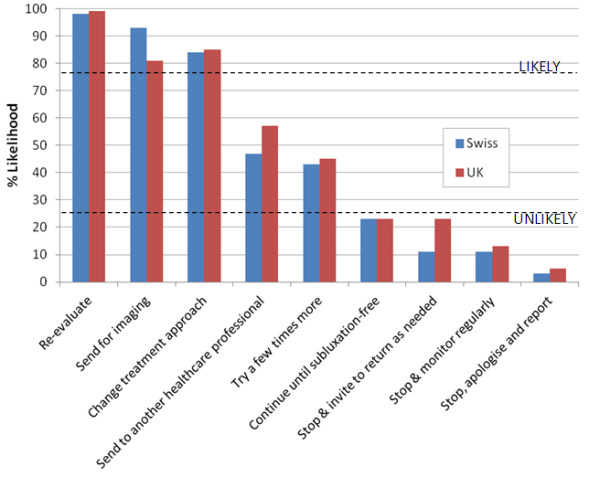
**Clinical management options in response to case scenario 3.** Likelihood of nine given clinical management options being followed in response to *Case Scenario 3*: *A patient returns from the last treatment with a new distal pain* (*e*.*g*. *sciatica when he was treated only for localized LBP*, *or brachialgia when he was treated only for local neck pain*). The dotted lines demarcate a region of the chart outside of which management options were arbitrarily designated as ‘likely’ (upper line) or ‘unlikely’ (lower line) to be undertaken. In this instance, Swiss and UK chiropractors were likely to re-evaluate, send for imaging, and change the treatment approach; both groups were unlikely to continue the treatment until the patient is subluxation-free, stop and invite the patient to return as needed, stop and then monitor regularly or stop to apologise and report to the incident reporting system.

**Figure 4:**
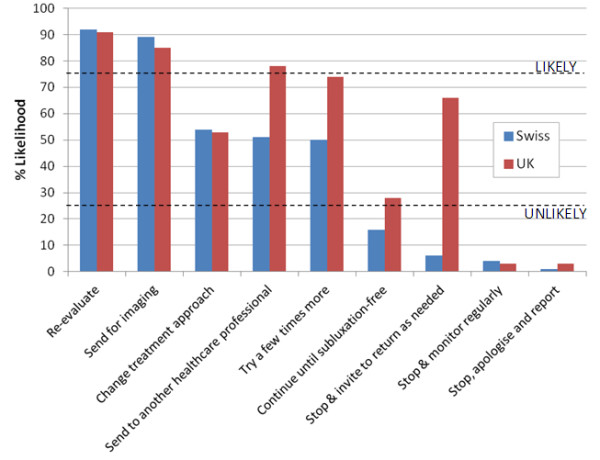
**Clinical management options in response to case scenario 4.** Likelihood of nine given clinical management options being followed in response to *Case Scenario 4*: *An elderly woman complains about immediate chest pain on inspiration after manual treatment directed to her thoracic spine*. The dotted lines demarcate a region of the chart outside of which management options were arbitrarily designated as ‘likely’ (upper line) or ‘unlikely’ (lower line) to be undertaken. Swiss and UK chiropractors were likely to re-evaluate, and send for imaging; UK chiropractors were likely to refer to another healthcare professional, Swiss chiropractors were unlikely to continue the treatment until the patient is subluxation-free; Swiss chiropractors were unlikely to stop and invite the patient to return as needed; both groups were unlikely to stop to monitor regularly or stop to apologise and report to the incident reporting system.

The likelihood of the nine clinical management options being undertaken in response to the four case scenarios is presented in Figures 1, 2, 3, and 4.

Scenario 1 (*a patient with non-specific low-back pain has not improved at all after 4–6 treatments),* suggests a case where treatment has not been successful. Figure 1 illustrates that under these circumstances:

•Swiss and UK chiropractors were likely to re-evaluate, send for imaging, and change the treatment approach.

•UK chiropractors were likely to refer to another healthcare professional.

•Both groups were unlikely to continue the treatment until the patient is subluxation-free.

•Both groups were unlikely to stop and then monitor regularly.

•Both groups were unlikely to stop to apologise and report to the incident reporting system.

•Swiss chiropractors were unlikely to stop and invite the patient to return as needed.

Additional comments highlighted that incident reporting may not be considered relevant in cases where there is a lack of progress (Table 2).

**Table 2:** Respondents’ comments on incident reporting considerations in the context of the case scenarios indicated

	
**Scenario 1. A patient with non-specific low-back pain has not improved at all after 4–6 treatments.**	**Scenario 2. A patient, who has a simple neck problem with no previous long-term problems, has now improved at least 80% and stayed at this level for a couple of weeks.**
‘*It wouldn*’*t have occured to me to report it to CPiRLS*, *in a case of* - “*not responded to treatment*” *unless they had had a reaction to treatment*”	‘*It makes no sense to me to stop treatment*, *apologise and report it to CPiRLS*, *which I consider more in the context of serious incidences and not lack of progress*.’
‘*There has been no incident why would one report to CPiRLS*?’	
‘*Report to CPiRLS but continue*’	
**Scenario 3. A patient returns from the last treatment with a new distal pain (e.g. sciatica when treated only for localized LBP, or brachialgia when treated only for local neck pain).**	**Scenario 4. An elderly woman complains about immediate chest pain on inspiration after manual treatment directed to her thoracic spine.**
‘*The case might possibly be considered to be material for CIRLS*.’	*‘I would still report it to the CPiRLS - but would continue with treatment, I don’t feel they are mutually inclusive.’*
*‘I would report to CPiRLS if the exacerbation was significant and sustained, but would rarely apologise unless I was clearly at fault.’*	*‘I would report the event to CPiRLS but I would not apologise as this suggests I have done something wrong.’*
*‘I would only report to the CIRLS when I made a bad judgement or didn’t access the findings properly.’*	*‘If it’s only a broken rib, with no pulmonary complication, I would not think of reporting to CIRLS so far, but maybe in the future I will.’*
*‘I don’t see reporting an incident on CPiRLS as necessarily being linked with stopping treatment.’*	*‘If (the patient’s pain) is extreme, and would not get better within a few days, I would report it to the CIRLS. Otherwise I would not.’*
*‘I would be most likely to re-examine the patient and if I felt I was the most likely cause of the increase in symptoms I would report to CPiRLS’*	

Scenario 2 *(a patient, who has a simple neck problem with no previous long-term problems, has now improved at least 80% and stayed at this level for a couple of weeks)* is a case where no further improvement is apparent. Figure 2 illustrates that under these circumstances:

•None of the management options suggested are categorised by our criteria as *likely* for either Swiss or UK chiropractors.

•Both groups were unlikely to stop to apologise and report to the incident reporting system.

•UK chiropractors were unlikely to try a few more times, or stop treatment and invite the patient to return as needed.

Again, additional comments highlighted that incident reporting may not be considered relevant in cases where there is a lack of progress (Table 2).

Scenario 3 *(a patient returns from the last treatment with a new distal pain (e.g. sciatica when treated only for localized LBP, or brachialgia when treated only for local neck pain)),* suggests the patient is getting worse following treatment. Figure 3 illustrates that under these circumstances:

•Swiss and UK chiropractors were likely to re-evaluate, send for imaging, and change the treatment approach.

•Both groups were unlikely to continue the treatment until the patient is subluxation-free.

•Both groups were unlikely to stop and invite the patient to return as needed.

•Both groups were unlikely to stop and then monitor regularly.

•Both groups were unlikely to stop to apologise and report to the incident reporting system.

Additional comments revealed differing views about the need to report such an incident, a perception that incident reporting is relevant only when the chiropractor is at fault, and also that incident reporting may not necessarily lead to cessation of treatment (Table 2).

Scenario 4 *(an elderly woman complains about immediate chest pain on inspiration after manual treatment directed to her thoracic spine),* suggests that treatment has caused injury. Figure 4 illustrates that under these circumstances:

•Swiss and UK chiropractors were likely to re-evaluate, and send for imaging.

•UK chiropractors were likely to refer to another healthcare professional.

•Swiss chiropractors were unlikely to continue the treatment until the patient is subluxation-free.

•Swiss chiropractors were unlikely to stop and invite the patient to return as needed.

•Both groups were unlikely to stop to monitor regularly.

•Both groups were unlikely to stop to apologise and report to the incident reporting system.

Additional comments revealed the view that incident reporting is only relevant in relatively extreme cases of injury or fault, and also that reporting does not necessarily lead to treatment being stopped (Table 2).

### Demographic differences in respect of case scenario responses

When we explored combined data from the Swiss and UK groups across all four clinical case scenarios, we identified a significant difference in the management option “to re-evaluate the patient with a view to establishing a better diagnosis” between practitioners with one to two years in practice and those with over 10 years in practice [the less experienced practitioners had a mean score of 3.18 (SD = .79) and the more experienced clinicians had a mean score of 3.49 (SD = .71) (p = 0.014)]. No other statistically-significant differences were observed in respect of age, college of graduation, years in practice or practice-setting. However, we identified numerous statistically-significant differences, for combined Swiss and UK data, between males and females for some of the management decisions for Scenarios 1–4:

With Scenario 1 *(a patient with non-specific low-back pain has not improved at all after 4–6 treatments),* female practitioners were significantly more likely to:

•send the patient for diagnostic imaging.

•send the patient for a second opinion to another healthcare professional but keep on monitoring their condition (see Table 3).

**Table 3:** **Significant gender differences in management decisions for scenario 1 *(a patient with non-specific low-back pain has not improved at all after 4–6 treatments)***

**Management choice**	**Males mean (SD)**	**Females mean (SD)**	**P value**
“I would send the patient for a second opinion to another healthcare professional but keep on monitoring their condition”	3.01 (.73)	3.24 (.74)	0.003
“I would send the patient for diagnostic imaging”	3.02 (.70)	3.24 (.66)	0.003

With Scenario 2 *(a patient, who has a simple neck problem with no previous long*-*term problems*, *has now improved at least 80*% *and stayed at this level for a couple of weeks*), female practitioners were significantly more likely to:

•re-evaluate the patient with a view to establishing a better diagnosis.

•send the patient for diagnostic imaging.

•change their treatment approach and use another technique.

•send the patient for a second opinion to another healthcare professional but keep on monitoring their condition.

•encourage the patient to continue the treatment until their spine is subluxation-free.

•stop the treatment, apologise and report the event to the chiropractic reporting and learning system (See Table 4).

**Table 4:** **Significant gender differences in management decisions for scenario 2 *(a patient, who has a simple neck problem with no previous long-term problems, has now improved at least 80% and stayed at this level for a couple of weeks)***

**Management choice**	**Males mean (SD)**	**Females mean (SD)**	**P value**
“I would encourage the patient to continue the treatment until their spine is subluxation free”	1.92 (.97)	2.23 (.95)	0.002
“I would change my treatment approach and use another technique”	2.76 (.80)	3.03 (.78)	0.002
“I would re-evaluate the patient with a view to establishing a better diagnosis.”	2.74 (.81)	2.98 (.79)	0.01
“I would send the patient for a second opinion to another health care professional but keep on monitoring their condition.”	2.13 (.68)	2.30 (.73)	0.04
“I would send the patient for diagnostic imaging.”	2.01 (.69)	2.21 (.72)	0.03
“I would stop the treatment, apologize and report the event to the chiropractic reporting and learning system”	1.38 (.49)	1.52 (.54)	0.03

With Scenario 3 (*a patient returns from the last treatment with a new distal pain* (*e*.*g*. *sciatica when treated only for localized LBP*, *or brachialgia when treated only for local neck pain*)), female practitioners were significantly more likely to:

•change their treatment approach and use another technique.

•send the patient for a second opinion to another healthcare professional but keep on monitoring their condition.

•encourage the patient to continue the treatment until their spine is subluxation-free (See Table 5).

**Table 5:** **Significant gender differences in management decisions for scenario 3 *(a patient returns from the last treatment with a new distal pain (e.g. sciatica when treated only for localized LBP, or brachialgia when treated only for local neck pain)***

**Management choice**	**Males mean (SD)**	**Females mean (SD)**	**P value**
“I would encourage the patient to continue the treatment until their spine is subluxation free”	1.72 (.90)	1.93 (.90)	0.03
“I would change my approach and use another technique”	3.03 (.67)	3.21 (.71)	0.01
“I would send the patient for a second opinion to another healthcare professional but keep on monitoring their condition”	2.52 (.69)	2.78 (.72)	0.001

With Scenario 4 (*an elderly woman complains about immediate chest pain on inspiration after manual treatment directed to her thoracic spine*), female practitioners were significantly more likely to stop the treatment, but tell the patient that they are welcome to return if they feel the need (Table 6).

**Table 6:** **Significant gender differences in management decisions for scenario 4 *(an elderly woman complains about immediate chest pain on inspiration after manual treatment directed to her thoracic spine)***

**Management**	**Males mean (SD)**	**Females mean (SD)**	**P value**
“I would send the patient for a second opinion to another healthcare professional but keep on monitoring their condition”	2.91 (.88)	3.21 (.82)	0.003
“I would send the patient for diagnostic imaging”	3.21 (.81)	3.43 (.71)	0.011
“I would stop the treatment, but tell the patient that she is welcome to return if she feels the need”	1.95 (.87)	2.20 (.82)	0.02

No specific management options were identified where male practitioners were significantly more likely to agree than females.

### Safety dimensions

**Figure 5:**
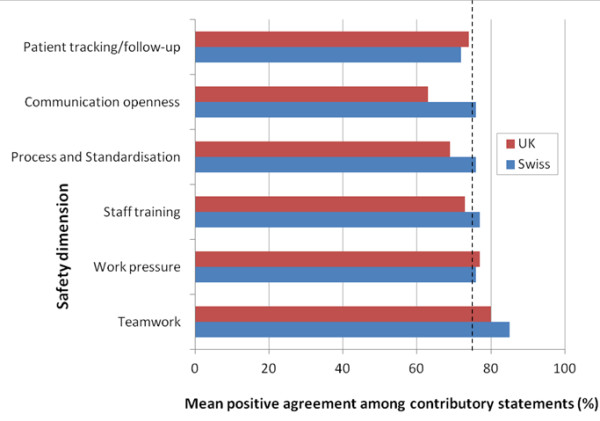
**Agreement with safety dimensions.** Degree of positive agreement among respondents with respect to six safety dimensions. A score greater than 60% but less than 75% was arbitrarily considered to indicate that respondents were moderately positive about the given safety dimension. A score of 75% (indicated by the dotted line), or greater, was arbitrarily considered to indicate that respondents were highly positive about the given safety dimension. Thus, it was established in this study that Swiss chiropractors were moderately positive about patient tracking/follow-up, and highly positive about all other safety dimensions. UK chiropractors were highly positive about work pressure and teamwork and moderately positive about all other safety dimensions.

The level of agreement of respondents with respect to the six safety dimensions measured is shown in Figure 5. This illustrates that:

•Swiss and UK chiropractors were highly positive about teamwork and work pressure as they relate to safety culture.

•Swiss chiropractors were highly positive about staff training, process and standardisation, and communication openness as they relate to safety culture.

•Swiss chiropractors were moderately positive about patient tracking/following-up as it relates to safety culture.

•UK chiropractors were moderately positive about staff training, process and standardisation, communication openness and patient tracking/following-up as it relates to safety culture.

## Discussion

### Case scenarios

For the case scenarios presented, there was a general trend that chiropractors were *unlikely* to stop treatment but were *likely* to re-evaluate, reflect on the diagnosis and alter the treatment approach.

For Scenario 1, where improvement has not been achieved after 4–6 treatments, some practitioners opted to cease treatment. However, there is a strong case to continue here despite the apparent lack of progress since NICE evidence-based guidance recommends up to 9 treatments over 12 weeks for persistent, non-specific low-back pain [7]. Reporting this scenario as a safety incident was found to be an unlikely course of action and two comments highlighted that such a case is not viewed as one that would prompt an incident report. However, one respondent commented that they would report the incident and also continue treatment. This revealed an attitude which recognises that harm and blame are not necessary triggers for an incident report, and perhaps that sharing an apparent lack of treatment success with colleagues may have learning value. We recognise the need for practitioners to be clear about what is meant by ‘safety incident’ and what should be reported; an issue that has been clearly identified by others as a barrier to incident reporting [8]. However, as far as CPiRLS and CRLS are concerned, there is guidance but no rules about what does and does not constitute a reportable incident. Use of these systems as general learning and care improvement tools is to be encouraged, particularly during a period when chiropractic incident reporting is a relatively new process and still becoming integrated into usual practice.

Scenario 4 suggested rib injury, such as a fracture or costo-chondral sprain, with osteoporosis as a possible risk factor. There is a strong argument for such an incident to be reported because patient injury occurred and because reflection on the detailed circumstances of the case, shared with colleagues, might serve to minimise the risk of such an occurrence happening elsewhere. However, incident reporting was found to be an unlikely option and comments revealed that this may be due to a perceived connection of reporting with guilt and error, as has been identified with other healthcare reporting initiatives [8], or only warranted in extreme cases. UK chiropractors appeared more likely to refer the patient in this case. This was presumably in the context of the injury rather than the original complaint since they also appeared much more likely to stop treatment and invite the patient to return as needed. This was supported by the comment:

‘In this scenario I would re-exam and most likely refer her back to her GP for further examination/diagnostic imagery. Treatment would stop until the results were in. Treatment would then commence depending on these results, perhaps this area would either be left or Activator technique utilised. Other areas of complaint would continue to be treated.’

### Gender differences in respect of case scenario responses

The fact that men and women have differing perceptions of risk is well documented with women having a lower propensity towards risky choices in most domains of life [9]. It is perhaps not surprising, therefore, that we identified gender differences in chiropractors’ management of potentially risky clinical scenarios. In our study, female chiropractors were more likely to refer patients for another opinion, and hence mitigate for risk, in three of the four scenarios. They were also more likely to stop treatment in two of the scenarios. However, interestingly, only in the case of Scenario 2 were they were more likely to report the incident to CPiRLS/CRLS. This may suggest that incident reporting is not viewed as an effective risk reduction measure; a finding observed in other studies [8].

### Safety dimensions

Our questionnaire also prompted chiropractors to reflect on safety culture and it was encouraging to note the positivity towards all workplace safety culture dimensions. Communication openness and professional collaboration are known to help prevent critical incidents [10] so the fact that Swiss respondents were highly positive and UK respondents moderately positive about communication openness, and both groups were highly positive about teamwork, bodes well. Respondents’ comments highlighted a number of contributory factors important for patient safety in the clinic environment, for example:

‘Regular meeting with the whole team helps to keep up with the quality and safety’.

However, some respondents also highlighted the fact that a high proportion of chiropractors (33% of UK respondents and 48% of Swiss respondents in our study) work alone, limiting opportunities for fostering a safety culture through activities such as teamwork.

### Limitations and further study

Caution should be exercised when drawing general conclusions about the UK chiropractic profession from the findings presented in this article since the response rate of 31% means the respondent group may not comprise a representative sample. We did not investigate, further, the reasons for this low response rate.

While we determined the demographic characteristics of the respondents, we do not know how well these characteristics represent those of the entire chiropractic populations in Switzerland and the UK.

While there were some apparent differences between respondents from Switzerland and those from the UK, we did not statistically analyse these possible differences.

Further studies specifically designed to investigate the attitudes towards and the barriers to using online chiropractic safety incident reporting and learning systems are now required in order to develop measures to increase participation.

## Conclusions

•This study prompted chiropractors to reflect on aspects of clinical risk.

•Swiss and UK chiropractors tend to manage potentially risky clinical scenarios by re-evaluating their care and changing their approach.

•Safety incident reporting to an online system is currently an unlikely course of action, probably due to previously recognised barriers, although Swiss and UK chiropractors are positive about local communication and openness which are important tenets for safety incident reporting.

•Barriers to the use of safety incident reporting systems need to be addressed in order to encourage wider use of the existing systems.

•A significant proportion of Swiss and UK chiropractors practice in a single-handed environment. We suggest that single-handed practitioners have most to gain from participation in a national safety incident reporting and learning system.

•Female chiropractors appear to be more risk-averse than male chiropractors.

•Positivity towards key aspects of clinic safety indicate a developing safety culture within the Swiss and UK chiropractic professions.

## Competing interests

The authors declare that they have no competing interests.

## Authors’ contributions

MW instigated this study and designed the first draft of the questionnaire. All authors provided general expertise on the topic and critical comments on the draft questionnaire. MW collected the questionnaire data and collated the findings, CP advised on and undertook the statistical analysis and RF drafted the manuscript. All authors reviewed the manuscript, provided critical comments and approved the final version. All authors read and approved the final manuscript.
